# The structurally constrained protein evolution model accounts for sequence patterns of the LβH superfamily

**DOI:** 10.1186/1471-2148-4-41

**Published:** 2004-10-22

**Authors:** Gustavo Parisi, Julián Echave

**Affiliations:** 1 Centro de Estudios e Investigaciones, Universidad Nacional de Quilmes, Roque Saenz Peña 180, B1876BXD Bernal, Argentina

## Abstract

**Background:**

Structure conservation constrains evolutionary sequence divergence, resulting in observable sequence patterns. Most current models of protein evolution do not take structure into account explicitly, being unsuitable for investigating the effects of structure conservation on sequence divergence. To this end, we recently developed the Structurally Constrained Protein Evolution (SCPE) model. The model starts with the coding sequence of a protein with known three-dimensional structure. At each evolutionary time-step of an SCPE simulation, a trial sequence is generated by introducing a random point mutation in the current coding DNA sequence. Then, a "score" for the trial sequence is calculated and the mutation is accepted only if its score is under a given cutoff, *λ*. The SCPE score measures the distance between the trial sequence and a given reference sequence, given the structure. In our first brief report we used a "global score", in which the same reference sequence, the ancestral one, was used at each evolutionary step. Here, we introduce a new scoring function, the "local score", in which the sequence accepted at the previous evolutionary time-step is used as the reference. We assess the model on the UDP-N-acetylglucosamine acyltransferase (LPXA) family, as in our previous report, and we extend this study to all other members of the left-handed parallel beta helix fold (LβH) superfamily whose structure has been determined.

**Results:**

We studied site-dependent entropies, amino acid probability distributions, and substitution matrices predicted by SCPE and compared with experimental data for several members of the LβH superfamily. We also evaluated structure conservation during simulations. Overall, SCPE outperforms JTT in the description of sequence patterns observed in structurally constrained sites. Maximum Likelihood calculations show that the local-score and global-score SCPE substitution matrices obtained for LPXA outperform the JTT model for the LPXA family and for the structurally constrained sites of class *i *of other members within the LβH superfamily.

**Conclusion:**

We extended the SCPE model by introducing a new scoring function, the local score. We performed a thorough assessment of the SCPE model on the LPXA family and extended it to all other members of known structure of the LβH superfamily.

## Background

Protein structure is more conserved than protein sequence during molecular evolution [1-3]. Remote homologous proteins constitute an extreme example of sequence divergence where proteins with similar function and no apparent sequence similarity present almost the same fold [4]. However, protein sequences are far from being random. Rather, they are selected through evolution in such a way that functional constraints modulate sequence variability. Usually, only a few residues are directly related to the protein function. However, these residues must maintain adequate spatial relationships for the protein to remain functional, so that the whole 3D structure is conserved. In turn, structure conservation constrains sequence variability in such a way that residue substitution does not disturb the overall 3D structure of the protein. This results in emergent non-random sequence patterns.

The restrictions imposed by the environment of a given protein site onto its pattern of amino acid substitutions have been largely discussed [2,5-9]. Briefly, highly constrained positions are more conserved. Furthermore, each site has a biased composition related to its structural environment. Models of protein evolution that take this into account outperform other, simpler, models [10-13]. Recently, a number of models of protein evolution have been developed that take explicit account of protein structure, stability, and/or foldability [14-22]. Even though such models have not been used yet for phylogenetic inference purposes, they are useful to gain insight into the detailed mechanism of protein evolution. Noteworthy, some of these models have been able to reproduce quantitatively observed amino acid substitution patterns [12,14,23].

To study how protein structure conservation modulates sequence divergence, we recently developed the Structurally Constrained Protein Evolution (SCPE) model [14]. The starting point of an SCPE simulation is the coding-sequence of a protein of known three-dimensional structure, which we shall call the "ancestral sequence". At each evolutionary time-step, a new "trial sequence" is generated by random mutation, at DNA level, of the "current sequence" (accepted at the previous time-step). Then, the trial DNA is translated using the universal genetic code and a "score" that estimates the protein structure perturbation introduced by the mutation is evaluated. The trial sequence is accepted, becoming the new current sequence, only if its score is below a certain "cut-off", *λ*, that measures the amount of structural perturbation allowed by natural selection. In this way, for *λ *= 0 only synonymous mutations are accepted, whereas for *λ *~ ∞ all mutations are accepted. The procedure is repeated until a desired number of mutations are reached. In the present work the DNA is mutated using the Jukes-Cantor model, so that each nucleotide substitution occurs with the same probability.

The model depends on one parameter, the cut-off *λ*, that must be fit by comparison to actual sequence data. Different properties could be used to fit the cut-off. As we will show below, the model is quite robust with respect to the property used. Therefore, we have used the simplest way, which is to fit *λ *such that the acceptance rate, *ω*, inferred for actual sequences is reproduced. The acceptance rate is the probability that an amino acid mutation is accepted. Thus, it can be estimated by the ratio between the number of amino acid substitutions (accepted mutations) and the total number of trial amino acid mutations. The acceptance rate has been extensively used to characterize the strength of the selective pressure under which proteins evolve [24-27]. If all mutations were neutral they would be accepted and *ω *would be 1. In general the *ω *values are usually below 0.5 due to the deleterious effects of most amino acid mutations [28]. In proteins under very strong selective pressure *ω *can take values very close to zero.

One of the main factors determining the quality of the SCPE model is the scoring function. Given the structure of the ancestral protein, which we assume constant throughout the simulation, the score of a given trial sequence is defined as the RMSD between the mean-field energy profile of the trial sequence and that of a reference sequence. In our previous work, the same reference sequence, the ancestral one, was used for each time-step. Therefore, the score of each trial sequence was a measure of the dissimilarity between the trial sequence and the ancestral sequence, given the structure. Such a score depends only on the trial sequence and the ancestral sequence, but not on the particular sequence mutated to obtain the trial. Hence, it does not depend on the precise evolutionary path between the ancestral and the trial. Therefore, this will be called from now on "global score".

Even though the global score has been proved to be very good at reproducing the sequence patterns of a test case, it also shows some problems. Mainly, at the beginning of a simulation most mutations fall below the optimum cut-off. This results in too high values of the acceptance rate. Only after about 5% of the sites have been substituted, the cut-off is purifying enough to reproduce the acceptance rate inferred for the actual family. From a more qualitative point of view, since at the beginning of global-score simulations almost all mutations are accepted, erroneous amino acids, which are not found in the natural sequences of the family studied, can be introduced with relatively high probability during the first few steps of a global-score simulation. We shall see below that these are unwanted artefacts of the global-score SCPE simulations.

To tackle the problems described in the previous paragraph, in this paper we introduce a "local score", in which the reference sequence for a given trial is that accepted in the previous evolutionary time step, the current sequence, rather than the ancestral one. Thus, the local score measures the mutational perturbation introduced in a given time-step, rather than the global difference between the trial and ancestral sequences.

**Figure 1:**
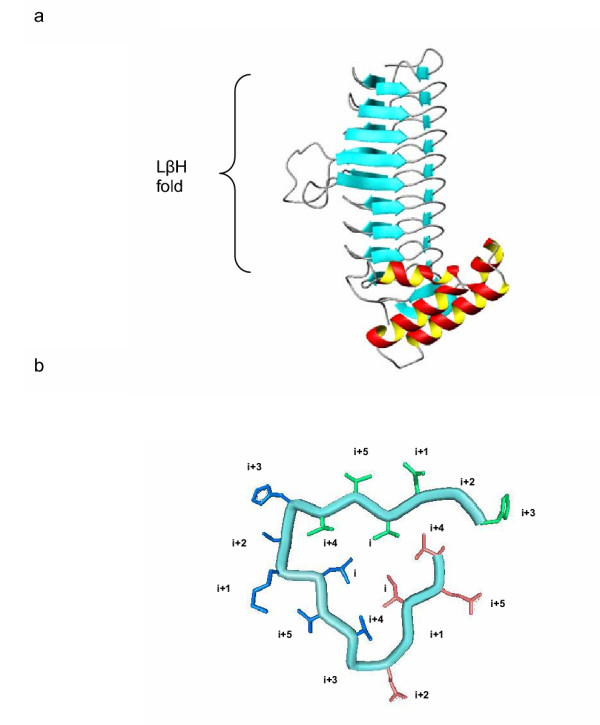
(a) Structure of the UDP-N-acetylglucosamine acyltransferase (LPXA). This protein forms a Left-handed parallel β Helix (LβH). (b) Detail of one coil of the helix. Each coil is formed by three hexapeptides (shown in different colours). Note that hexapeptide positions *i *and *i+4 *point towards the inside of the prism whereas the other positions point outwards.

The new approach is compared with the previous one on the same test system studied before: UDP-N-acetylglucosamine acyltransferase (LPXA) from *Escherichia coli*. A portion of this protein presents a left-handed parallel beta helix (LβH), a fold generally associated with transferase activity and broadly distributed in different taxons [29-31] (see Figure 1a). All the LβH proteins contain a hexapeptide-repeat motif which is closely related with the topology of the fold (Figure 1b). This superfamily is characterized by the high conservation of the fold that contrasts with an elevated sequence and functional divergence.

We shall show below that when the local score is used, the acceptance rate averaged over independent runs does not depend on the amount of divergence from the ancestral sequence. Furthermore, no erroneous amino acids are accepted during the simulations. Thus, these artefacts of the global-score simulations are absent when the new scheme is used. To further compare both schemes, other properties were analysed. Specifically, we evaluated and compared structure conservation, entropy profiles, amino acid distributions, and substitution matrices. We show that SCPE simulations that use the LPXA from *E. coli *as ancestral sequence can be used to estimate site-dependent amino acid substitution matrices [32,33] which outperform the usually used JTT model [34]. Moreover, we consider the applicability of the SCPE substitution matrices obtained from LPXA simulations to other protein families which adopt the LβH fold.

## Results and discussion

### Acceptance rates

**Figure 2:**
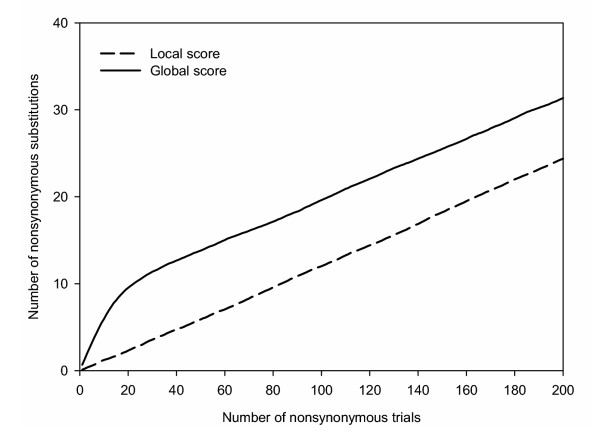
Number of nonsynonymous (amino acid) substitutions observed as a function of the number of nonsynonymous (amino acid) mutation trials for local-score and global-score SCPE simulations. Results are averaged over 300 independent runs. Note that global-score simulations present a definite change in slope (acceptance rate *ω*) between the first steps of the simulations and longer times. In contrast, local-score simulations present a constant slope (acceptance rate *ω*).

In Figure 2 we show the number of nonsynonymous substitutions versus the number of nonsynonymous mutations averaged over several independent simulations. Note that nonsynonymous substitutions (mutations) at DNA level are amino acid substitutions (mutations) at protein level. The slope of each plot is the acceptance rate *ω*. Figure 2 shows that for the global-score case, *ω *decreases from *ω *= 1 when the simulation begins to a constant asymptotic value *ω *< 1 for longer times. In an actual case, such behaviour could be due to a sequence that for some reason is particularly robust with respect to mutations. In the present case, however, this is an unintended artefact of our model. It happens because the global score of the mutations introduced in the first steps of a simulation lie below the global cut-off, no matter how nonconservative the mutation is. Thus, at the beginning of a global-score simulation almost all amino acid mutations are accepted, leading to an acceptance rate *ω *= 1. Furthermore, clearly wrong amino acids, that will irreversibly upset the structure, can be introduced. In contrast, the local-score simulations display a constant average *ω*, which we think is more consistent with a neutral model, such as SCPE, with constant selection pressure *λ*. We should mention that despite the constancy of the average *ω*, the acceptance rate *ω *of a single simulated run changes from sequence to sequence. This is expected, since any substitution at a given site changes the scores of the sites that are in contact with it in the 3D structure. This could account for features such as overdispersion of the molecular clock and rate-shifts in substitution rates (heterotachy).

### Determination of optimal *λ*

**Figure 3:**
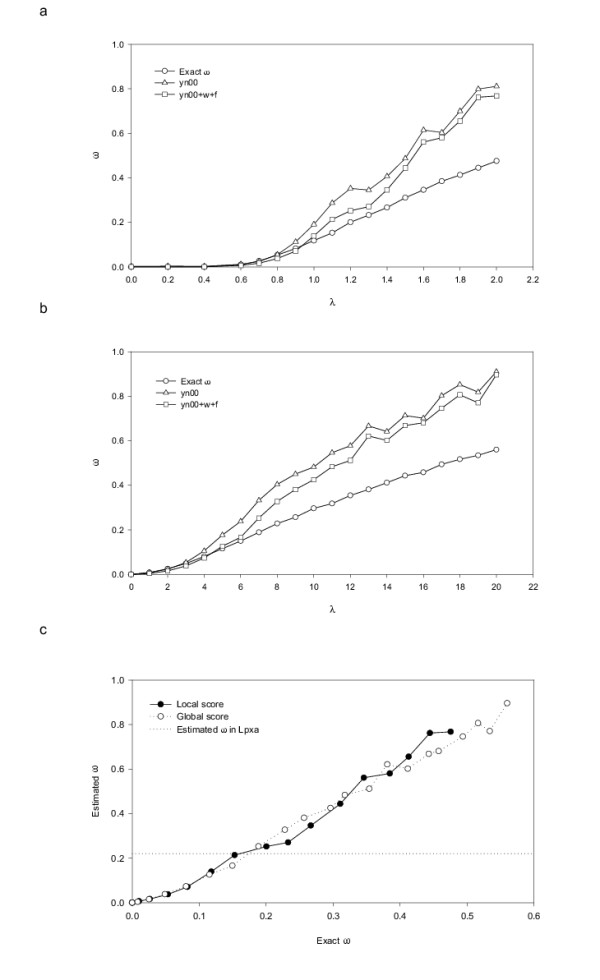
Inferred and calculated acceptance rates of data sets simulated with SCPE. (a) local-score simulations. (b) global-score simulations. yn00 and yn00+w+f are two different methods to infer acceptance rates included in PAML (see Methods). The average acceptance rate inferred in the LPXA reference alignment (obtained with yn00+w+f) is 0.2246 ± 0.11. Using this value in (a) and (b) the optimal local and global *λ *obtained are 1.10 and 7.00, respectively. In (c) we plot the *ω *inferred using yn00+w+f versus the calculated value for SCPE runs from (a) and (b). The value inferred for the observed LPXA family is shown as a dotted line. Using this value the optimal *ω *for local score is 0.15(0.12–0.27) and for global score is 0.19(0.12–0.26).

As discussed in Methods, we have chosen to determine the optimum value of the SCPE model parameter *λ *so that the acceptance rate of the simulations matches that inferred from actual sequences. For the SCPE simulations, the value of *ω *is easily estimated by just counting the number of amino acid substitutions accepted throughout the simulations and dividing by the number of trial amino acid mutations. For the reference alignment, however, one has to estimate *ω *using some inference method. These methods tend to overestimate the actual *ω*. This can be seen in Figure 3, where we show two different *ω *inferences for a set of SCPE simulations as a function of the cut-off *λ*, together with the value calculated by counting the proportion of accepted mutations (see Methods). The inferences were made with the module yn00 of PAML [35] as explained in Methods. It is worthwhile to note that the inferred *ω *departs from the calculated *ω *as *λ *increases. This behaviour is expected since, for a given number of mutations, for larger *λ *there are more accepted nonsynonymous substitutions, which results in loss of sequence signal.

Using the method yn00+w+f, which best estimates the calculated *ω*, we obtained the *ω *of the reference alignment of 25 sequences homologous to the UDP-N-acetylglucosamine acyltransferase from *Escherichia coli *(LPXA reference alignment). The average *ω *for this alignment is 0.22. Using this value in Figure 3a and 3b the optimal values of *λ *obtained are 1.10 and 7.00 for local and global score, respectively.

We note here that the optimal *λ *values for local and global score are very different. Thus, for the sake of comparison, we take advantage of the one-to-one relationship between *λ *and *ω*, shown in Figures 3a and 3b, and use the calculated acceptance rate *ω *instead of *λ *as model parameter. In Figure 3c we plot the inferred *ω *versus the calculated *ω *for local-score and global-score simulations. Using this plot and the inferred *ω *value for the LPXA reference alignment, *ω *= 0.22 ± 0.11 (0.11 is the standard deviation of *ω*), we calculate an optimal *ω *of 0.15 (0.12–0.27) for local score and 0.19 (0.12–0.26) for global score.

### Assessment of structure conservation

It is important to assess if the SCPE models are able to preserve protein structure. To this end we used THREADER 3 to analyze the percentage of sequences that recognize the correct structure using different models. Results are shown in Table 1. Clearly, JTT is unable to conserve structure even for relatively low amounts of divergence: at Ka = 0.28 only 20% of sequences obtained from JTT simulations recognize the correct structure. In contrast, a significant proportion of sequences simulated with SCPE recognize the correct structure even after long simulations of 1.7 substitutions per site: 62% for local-score SCPE and 39% for global-score SCPE.

**Table 1:** Evaluation of structure conservation. The table shows the percentage of output sequences that recognize correctly the LβH fold for local-score SCPE, global-score SCPE, and JTT for two different amounts of amino acid substitutions per site (Ka).

	Amount of Divergence	
Model	Ka = 0.28	Ka = 1.7
Local-score SCPE *λ *= 1.10, *ω *= 0.15	87%	62%
Local-score SCPE *λ *= 8.00, *ω *= 0.92	19%	4%
Global-score SCPE *λ *= 7.00, *ω *= 0.19	68%	39%
Global-score SCPE *λ *= 90.00, *ω *= 0.95	8%	0%
JTT	20%	0%

When both SCPE schemes are compared, Table 1 shows that local-score simulations perform better than global-score ones. This result is counterintuitive, because one might expect, *a priori*, that in the long term the global-score would be better at conserving structure than the local-score, since in the later case the reference sequence is reset at each step so that it would be easier to lose memory of the ancestral protein. One of the reasons of the global-score SCPE being worse at conserving structure could be the erroneous amino acid substitutions introduced at the beginning of the simulations (see above). To gain more insight into this issue, further work involving much longer simulations would be needed. However, for long enough evolutionary time it is not longer reasonable to assume that structure remains constant. In this limit, any model based on assuming structural conservation will break down.

### Entropy profiles

**Figure 4:**
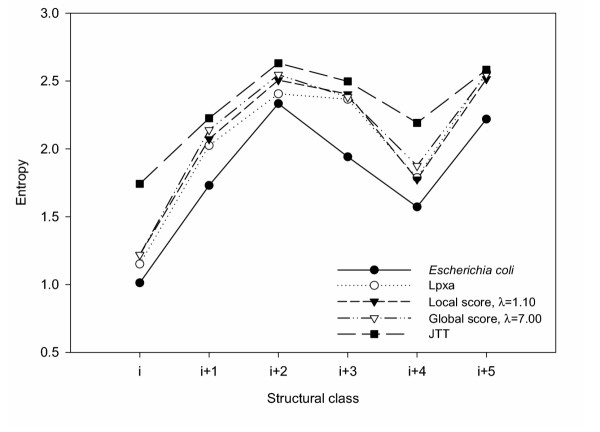
Entropy profiles. Each structural class corresponds to a particular position in the hexapeptide motif found in the LβH proteins. Structural classes *i *and *i+4 *are the most conserved while the other classes present a more variable composition. SCPE profiles correspond to equilibrium amino acid distributions (see Methods). The SCPE parameters were fit to the minimum of the entropy error (see Figure 5). The profile obtained from JTT simulations of 0.28 substitutions per site is shown for comparison.

To evaluate the capacity of the SCPE model to reproduce the sequence patterns found in the LPXA family, the variability of each site was analysed. The different protein positions were accumulated into 6 structural classes. For each class, we calculated the entropies corresponding to the equilibrium distributions of SCPE models. These entropies represent the average structural constraints of each structural class and do not depend on simulation time. SCPE entropy profiles are compared with those obtained from the reference alignment of the LPXA family. One could argue that these are not only determined by structure, but also contain historical information. However, since we are accumulating over several sites of the same class, which would have independent evolutionary histories, we expect such information to be somewhat averaged out. For the sake of comparison we also calculated the entropy profiles of JTT simulations of 0.28 amino acid substitutions per site (see Methods). The resulting entropy profiles are shown in Figure 4. It can be seen from this figure that both, the local-score and the global-score schemes reproduce very well the variability pattern of the LPXA family. Also, in Figure 4 we show that simulations performed using the JTT model produce less accurate results, especially for the most conserved (low entropy) structural classes *i *and *i+4*.

**Figure 5:**
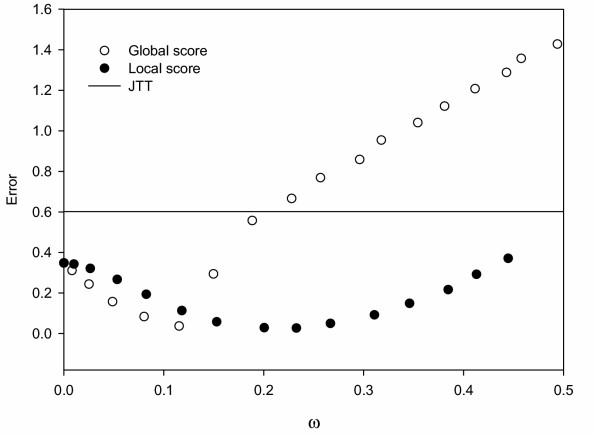
Error in entropy profiles between observed and equilibrium SCPE amino acid probability distributions versus calculated acceptance rate. Results for local-score and global-score SCPE simulations are shown, together with those obtained from JTT simulations of 0.28 amino acid substitutions per site.

To further study the effect of varying the model parameter, we calculated an "error" which quantifies the difference between simulated and observed entropy profiles (see Methods). In Figure 5 this error as a function of *ω *is shown. Comparing Figures 5 and 3, we see that the *ω *for which the entropy error is minimum is consistent with the value at the optimum cut-off for both the local and the global score schemes.

### Probability distributions

**Figure 6:**
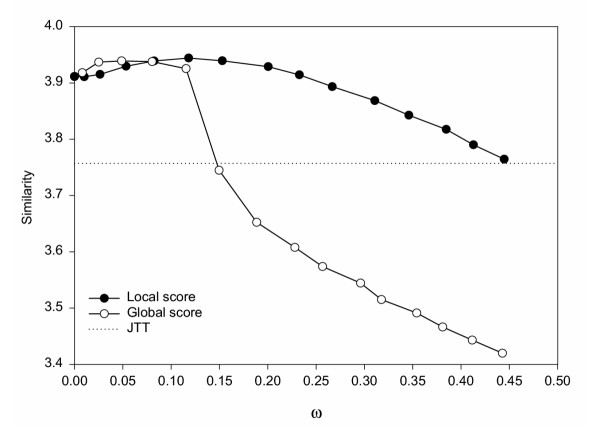
Similarity score between observed and equilibrium SCPE amino acid probability distributions versus calculated acceptance rate. Results for local-score and global-score SCPE simulations are shown, together with those obtained from JTT simulations of 0.28 amino acid substitutions per site. For *ω *= 0, the distribution is that obtained from the ancestral sequence, LPXA of Ecoli by grouping sites of the same class, since in this case no substitution is accepted and therefore it is impossible to obtain the SCPE substitution matrices. For SCPE we used equilibrium distributions, which do not depend on time. JTT results become worse for longer times (Ka>0.28).

Although entropy is commonly used to evaluate sequence conservation in an alignment [36-38] and to compare simulated data with natural sequences [39,40], it is not enough for a thorough assessment of the sequence pattern. An entropy value of 0 at a given site, for example, means that there is only one amino acid, but it could be any one out of twenty. Thus, to perform a more complete evaluation of the SCPE model, we looked into the amino acid probability distributions. To this end, we calculated a similarity score between the asymptotic SCPE distributions and those obtained from observed sequences. We used the similarity score used by Yona and Levitt to perform sequence profile-profile comparisons [41]. In Figure 6 we show the similarity score between observed and SCPE equilibrium amino acid distributions as a function of the calculated acceptance rate *ω*. We also show results for a simulation performed using the JTT model [34] of evolution. Overall, it can be seen that the local-score SCPE performs somewhat better than the global-score SCPE, and that both SCPE models clearly outperform JTT for a significant range of parameter *ω *around the optimum value.

A more detailed analysis shows that the maximum of the local-score plot corresponds to a *ω *= 0.12, that is in good agreement with the optimum cut-off determined from the acceptance rates, as explained previously. In contrast, for the global-score case the cut-off at the maximum of the similarity score plot is significantly below the optimum *ω *value previously obtained. This difference would be due to the wrong behaviour of the global-score scheme for small amounts of divergence (see Figure 2), which will affect the SCPE substitution pattern and, therefore, the amino acid probability distributions. The same behaviour, though less marked, is found in the plots of Figure 5.

Finally, it is interesting to note that the similarity score for *ω *= 0 is much better than JTT. Since *ω *= 0 corresponds to a simulation where no nonsynonymous substitutions are accepted, this is the score obtained using just the initial sequence. Memory of this sequence might favour the good agreement observed for SCPE. However, it is noteworthy that the actual agreement increases for *ω *> 0, showing that the good fit is not due exclusively to a memory effect. The substitution matrix assessment described in the next section should be less sensitive to memory effects.

### Substitution matrices

Even though it has long been recognized that substitution patterns are site-specific and depend on protein family, it is in general very difficult to estimate site-specific and family-specific substitution matrices due to a lack-of-data problem. As we reported previously, a possible strategy to overcome this obstacle is to obtain site-specific substitution matrices from SCPE simulations [12]. To further evaluate how the SCPE model is able to reproduce the substitution pattern of the LPXA family, a maximum likelihood analysis was used. SCPE runs were used to obtain a substitution matrix **Q**^*c *^for each structural class. Then, these matrices were used to calculate the maximum likelihood of the LPXA reference alignment using a given topology (see Methods).

**Figure 7:**
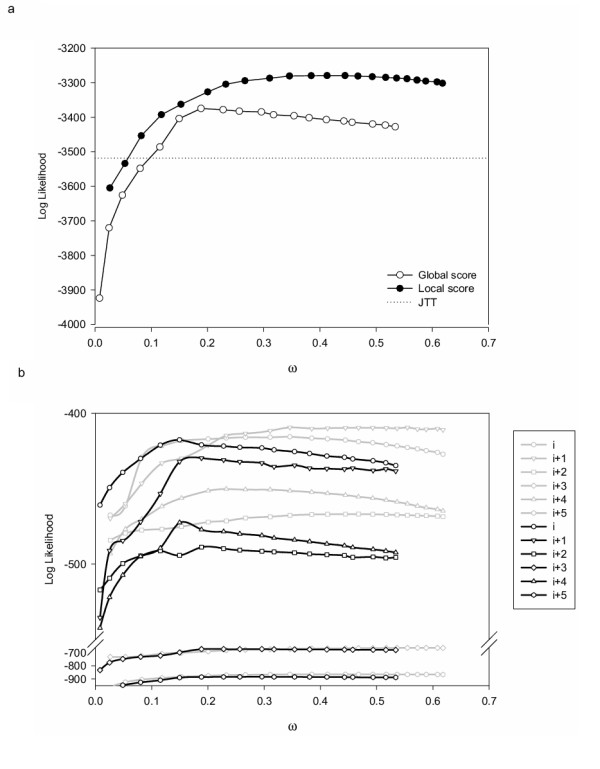
Maximum likelihood as a function of calculated acceptance rate. (a) Likelihood obtained using local-score and global-score SCPE substitution matrices as a function of *ω*. We also show the likelihood obtained using JTT. (b) Likelihood for the six structural classes using local-score and global-score SCPE matrices.

In Figure 7a we show the likelihood vs. *ω *plots obtained using local-score and global-score SCPE substitution matrices. The global-score likelihood peaks near *ω *= 0.18 in good agreement with the previous determinations, showing that it reproduces quite well the amino acid substitution patterns found in real sequences. The best *ω *of the local-score SCPE likelihood (see Figure 7a) corresponds to *ω *= 0.4, larger than that determined previously (Figures 3, 5, and 6). To understand this behaviour, we analysed the log likelihood components for each structural class, which are shown in Figure 7b. It is seen from this figure, that the local-score maximum likelihood peaks near *ω =* 0.4 mainly because of the contributions of structural classes *i+1*, *i+2*, *i+3*, and *i+5*, which, being the least structurally constrained sites, are not expected to be very well reproduced by SCPE. In contrast, for those sites that point towards the inside of the LβH helix, which are the ones the model should best describe (conserved classes *i *and *i+4*) the maximum likelihood peaks near *ω *= 0.2, in better agreement with Figures 3, 5, and 6. In the global-score case, from Figure 7b, the maximum likelihood plots for different classes behave more evenly: for all classes, the maximum likelihood peaks near *ω *= 0.15.

Figure 7a shows that for LPXA, local-score simulations lead to better substitution matrices than global-score ones. Inspection of Figure 7b reveals that this is mainly due to the local-score SCPE giving better results for sites i+4 and, to a lesser degree, i+2. Figure 7a also reveals that both, local and global, SCPE models outperform JTT (dotted line of Figure 7a) for almost the whole *ω *range studied. This is due to the fact that site-specific amino acid substitution patterns, especially for constrained structural classes *i *and *i+4*, are not well described by general models such as JTT.

### Other LβH families

As a further example of the applicability of the SCPE model, we considered other families of the LβH superfamily (see Table 2). We used the local-score and global-score schemes with the optimum cutoffs estimated using acceptance rates, as explained previously, to obtain site-dependent probability distributions and substitution matrices for the six different structural classes.

**Table 2:** LβH superfamily members studied.

**Gene name or synonym**	**Function**	**PDB ID**	**Number of sequences aligned**
LPXA	UDP-N-acetylglucosamine acyltransferase	1lxa	25
SATA	Streptogramin A Acetyltransferase	1kk6	48
LACA	Galactoside O-Acetyltransferase	1kru	43
CAT	Xenobiotic Acetyltransferase	1xat	39
DAPD	Tetrahydrodipicolinate-N-Succinlytransferase	1tdt	50
CAM	Carbonic Anhydrase	1qre	26
GLMU	N-Acetylglucosamine-1-Phosphate Uridyltransferase	1g97	50

**Figure 8:**
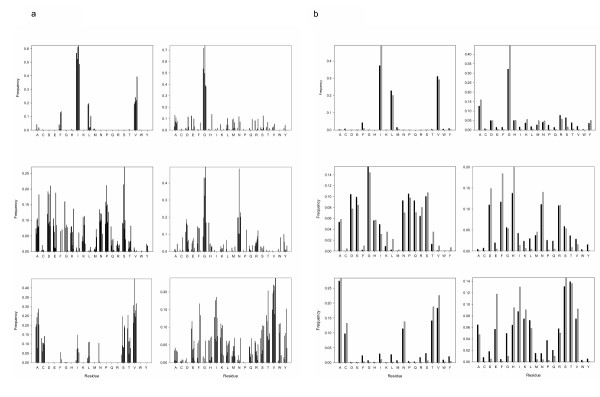
Amino acid frequency distributions for the hexapeptide sites. (a) 7 LβH families of Table 2. (b) Local-score (grey) and global-score (black) SCPE equilibrium distributions (see Methods).

In Figure 8 we show the probability distributions of the LβH families considered (Figure 8a) and the equilibrium distributions obtained using the local-score and global-score SCPE models (Figure 8b). It can be seen that both SCPE schemes perform quite well in reproducing the sequence pattern of our test system.

To test the substitution matrices, we performed maximum likelihood calculations on each family of Table 2. Since the models compared have the same number of parameters, they can be compared using Maximum Likelihood (ML) values obtained using a reasonable phylogenetic tree topology [42,43] (see Methods). In Table 3 we show the ML values per site for local-score SCPE, global-score SCPE, and JTT, applied to different sets of sites. Better models have larger ML values.

**Table 3:** Comparison of models on 7 families of the LβH superfamily. Logarithm of the Maximum Likelihood per site obtained with different models for the families studied. Better models lead to larger ML values. The three numbers reported for each case correspond to structural classes *i *and *i+4*, considered separately, and to the average over the six structural classes.

**Family**	**Local score**	**Global score**	**JTT model**
LPXA	-19.9	-20.0	-23.7
	-21.7	-22.7	-24.9
	-28.0	-28.1	-29.3

SATA	-16.2	-16.1	-18.7
	-20.1	-19.2	-20.2
	-25.2	-24.7	-25.9

LACA	-35.7	-34.2	-36.3
	-36.4	-35.0	-35.9
	-38.0	-37.0	-37.4

CAT	-13.8	-13.7	-16.4
	-15.8	-15.9	-17.4
	-19.3	-19.0	-19.8

DAPD	-17.0	-17.1	-19.6
	-22.0	-23.8	-23.2
	-18.7	-20.0	-19.4

CAM	-13.9	-14.1	-15.9
	-20.3	-18.5	-18.6
	-23.1	-21.2	-20.4

GLMU	-38.1	-38.0	-39.9
	-39.0	-38.1	-38.1
	-49.3	-47.8	-47.9

For LPXA, SCPE (both local and global) are clearly better than JTT for all sites considered. CAT and SATA behave similarly, though the advantage of SCPE over JTT is less marked. For other families, SCPE (local and global) is better than JTT for class *i *sites. For other structural classes there is no definite advantage of SCPE over JTT.

When comparing local-score SCPE with global-score SCPE one finds no definite advantage of either one over the other. For sites *i*, where the more meaningful results are expected, local and global give very similar results for all families except for LACA where global is better than local.

## Conclusion

We presented in full detail the Structurally Constrained Protein Evolution Model (SCPE), developed recently. We improved on our previous model by introducing a new scoring function. Our previous work was based on a "global" score, which measures how a trial sequence differs from the ancestral sequence in its ability to fit a reference structure assumed constant. In contrast, the "local" score measures the perturbation introduced by a given mutation with respect to the previously accepted sequence, rather than the ancestral one.

Both schemes, global and local, were compared in their ability to match the substitution patterns of the protein family LPXA. We performed a thorough assessment comparing structure conservation, entropy profiles, amino acid distributions, and substitution matrices. LβH proteins were found to be particularly suited for such a detailed characterization of the sequence pattern, because of the fact that most of their sites belong to one of only six different structural classes. Furthermore, these properties were studied as a function of the single parameter of the model: a cutoff that measures selection pressure against structural divergence. Finally, we applied the model to all other members of the LβH superfamily whose structure is known, extending previous studies performed only on the LPXA family.

In general, we found that the local-score SCPE behaves either similarly or better than the global-score scheme, depending on the property considered. Furthermore, for LPXA, and for sites of the structurally constrained class *i *of all other families studied, both SCPE models clearly outperform the widely used JTT model, showing the power of the SCPE model to account for substitution patterns conditioned by structural constraints.

Currently, we are using the SCPE model to investigate several issues important in protein evolution, such as overdispersion of the molecular clock, correlation between the evolution of different sites, and heterotachy. Also, we are testing the applicability of the SCPE model to other protein families, in order to assess its generality. Nevertheless, we should mention that since most protein families do not display the regularity of LβH proteins, it is more difficult to perform a detailed quantification of sequence patterns, which makes such tests at the same time more difficult and less demanding than the LβH superfamily.

## Methods

### Test system

The LPXA family belongs to a large and diverse group of proteins [31], the LβH (Left-handed parallel β Helix) superfamily. All the sequences of this superfamily contain an imperfect tandem-repetition of a hexapeptide motif [29]. This motif is typically described by [LIVMA]-X_3_- [ASCVTN]-X. The first position of the hexapeptide is called *i*, and the following *i+1*, *i+2*, up to *i+5*. The sequence forms a left-handed parallel β helix, forming an equilateral triangular prism [44] (Figure 1a). Each coil of the helix is formed by three hexapeptides. Equivalent positions of different hexapeptides fall into similar structural environments. Residues at positions *i *and *i+4*, for example, point towards the inside of the β helix (Figure 1b). Thus, each site of the hexapeptide pattern corresponds to a different structural class. In this study we did not analyse sites that are at loop regions. Also, the first and last coils of the β helix of LPXA were not considered, since the structural environments of sites in these coils are not exactly the same as those of the other coils. Although all the LβH members have a homo-trimeric active form, we only use the monomer form in this study. We also analyse other LβH families, which are summarized, with a brief description, in Table 2.

### SCPE score

The first step in the calculation of the SCPE score is the calculation of a profile of mean energies per position. In the present case we used the Cβ-Cβ potential of the program PROSA II [45]. The original coordinates of the ancestral sequence were modified in order to provide with Cβ coordinates to those residues without them. Thus, all the GLY residues were substituted for ALA residues and an adequate rotamer was chosen using the program SCWRL [46]. Later, the substituted ALA residues were converted back to the original GLY, keeping the Cβ coordinate of ALA to use when a GLY mutates to a residue with Cβ. Once the energy per position is obtained the score is calculated using:



where N is the length of the protein sequence, *E*_*mut*_*(p) *is the mean-field energy of position *p *in the trial (mutated) sequence and *E*_*ref*_*(p) *is the corresponding value of the reference sequence. The "global score" is calculated using the ancestral sequence as reference. The "local score" is calculated using the sequence accepted in the previous step in the simulation (i.e. the sequence that is mutated to obtain the trial).

### SCPE simulations

The ancestral sequence was the UDP-N-acetylglucosamine acyltransferase (LPXA) from *Escherichia coli*. The coordinates were obtained from the PDB database [47] (ID code 1lxa). The cutoff range covered was 0–2.00 with a step of 0.1 for local score and 0–20 with a step of 1.00 for global score. For each cutoff value we performed 300 independent simulations, each one of 2500 mutational steps.

### Sequence analysis

Using the LPXA from *Escherichia coli *as the reference protein, we recovered 25 homologous sequences using sequence similarity searches. This set constitutes the reference LPXA family. For each of the other members of the LβH superfamily for which at least one member has known structure, we used this member's sequence to characterize putative homologous proteins. See Table 2 for details. All the similarity searches were performed using the program BLASTP [48] at the NCBI server and the sequence alignments were obtained using Clustal X [49].

### Estimation of acceptance rates

To assess the optimal selective pressure in our SCPE simulations, we inferred the mean *ω *value in the homologous LPXA family. Also, we inferred the *ω *in our SCPE simulations for different cut-offs. All the *ω *inferences were made using the program yn00 from PAML [35]. We used options "w", which applies a weighting scheme between codons, and "f", which takes into account the codon frequencies of the data.

In the SCPE simulations, we also estimated *ω *directly by counting: *ω *is the ratio between the number of amino acid substitutions (accepted mutations) and the total number of amino acid mutation trials. We use "calculated", as opposed to "inferred" to designate the acceptance rates obtained in this way.

### Estimation of the amount of divergence

Some of the comparisons performed depend on the amount of divergence. For these cases, we estimated the average divergence of the LPXA family using the program PAML[50]. Maximum likelihood distances were estimated using the JTT model with the frequencies estimated from the data and a gamma distribution with 8 categories to estimate the relative rates (JTT+F+Γ). The average time calculated was Ka = 0.28 amino acid substitutions per site.

### Assessment of structure conservation

We evaluated whether sequences produced by evolutionary simulations using SCPE recognize the correct structure using THREADER 3 [51]. We considered the following schemes: local-score SCPE with *λ *= 1.10 (*ω *= 0.15); local-score SCPE with *λ *= 8.00 (*ω *= 0.92); global-score SCPE with *λ *= 7.00 (*ω *= 0.19); global-score SCPE with *λ *= 90.00 (*ω *= 0.95). To compare, we also ran simulations using JTT. For each model, we performed 50 independent runs of lengths Ka = 0.28 and Ka = 1.7 amino acid substitutions per site. For each sequence, structure recognition using THREADER 3 was performed. The ability of models to conserve structure was measured by the percentage of sequences which recognized correctly (Z-score > 2.7) the LβH fold.

### Substitution matrices

Site-specific replacement matrices are obtained straightforwardly by "counting" substitutions in SCPE simulations. For the test system considered, sites can be classified into *c *= 1,2,...6 site classes. Then, for each class we set up a matrix of counts: for *i *≠ *j*,  is half the number of mutational steps that result in either *i *→ *j *or *j *→ *i *amino-acid replacements at site class *c*, and  is the number of mutational steps for which amino acid *i *remains constant (*i *→ *i *replacement). Then, for each class, the matrix of substitution rates, **Q**^*c*^, is obtained using:



Given the rate matrices, **Q**^*c*^, the probability matrices are obtained using

**P**^*c *^= exp(*t***Q**^*c*^)

The vector of amino acid equilibrium frequencies of class *c *is, then, obtained with



Since there are some substitutions that do not occur during the simulations (very low probabilities), we have found it convenient to re-calculate each **Q**^*c *^using a pseudocounts procedure similar to that developed by Tatusov [52] as follows



where  and  are, respectively, the substitution matrix elements and equilibrium frequencies of a reference model. Here we used JTT [34] and *α *= 0.01. Accordingly, equilibrium frequencies were also corrected using



### Entropies and amino acid distributions

To study the sequence variability profile, we calculated the entropy for each structural class using:



where  is the probability of finding residue *i *at structural class *c*.

For SCPE, we used the equilibrium probabilities obtained from the substitution matrices, as described in the previous section. For the reference alignment, we grouped all columns of the same structural class together, counted the number of times each amino acid occurred in each class, and obtained the corresponding amino acid frequencies.

The difference between the entropy profiles obtained from the SCPE models, , and the profile of the observed reference family, , was quantified by the following "error" function:



To assess the similarity between the equilibrium SCPE amino acid distributions and those obtained from the reference alignment, we used the similarity score based on information theory proposed by Yona and Levitt [41]. The score is calculated by adding together the similarity scores of the six structural classes.

### JTT distributions and entropies

The equilibrium SCPE distributions and their corresponding entropies were compared with JTT distributions and entropies. In contrast to SCPE, the equilibrium JTT distribution does not depend on structural class. Therefore, instead of the equilibrium distributions, we chose to use the distributions and entropies from the alignment of sequences obtained from simulations with the JTT model. To this end, we performed 100 independent simulations using the JTT substitution matrix. The simulation length was set to the average number of substitutions obtained for the LPXA family (Ka = 0.28). We aligned the 100 output sequences, grouped all columns of the same structural class together, counted the number of times each amino acid occurred in each class, and obtained the corresponding amino acid frequencies.

### Maximum likelihood calculations

In order to assess the SCPE substitution patterns, we performed Maximum Likelihood (ML) calculations using the site-dependent SCPE substitution matrices, **Q**^*c*^. The maximum likelihood of a model, **Q**, given the data, *s*, for topology, *T*, is obtained by maximizing the probability *L *= Pr(*s*|*T*, **Q**).

For the SCPE model, the reference alignment was partitioned into 6 sub-alignments corresponding to the 6 structural classes. Using these sub-alignments and the corresponding SCPE **Q**^*c *^matrices, we calculated the maximum likelihood using PAML. In all cases a gamma distribution was used to take into consideration the rate heterogeneity among sites of the same class. Similarly, we performed ML calculations using the JTT substitution matrix with gamma distribution of rates (JTT+Γ), for each of the six structural classes. The ML values obtained for each class were added together to obtain the total ML, as was done with the SCPE models.

It has been shown that as long as the tree topology is reasonable, model comparison is robust with respect to variations in topology [43]. In the present case, topologies were obtained using the program FITCH [53] of PHYLIP 3.57c [54] with ML distances obtained using JTT with PAML.

All the models compared here have the same number of parameters. Therefore, models were compared by comparing ML values. One should note, however, that when models with different number of parameters are compared, one should use a statistic that takes explicit account the number of parameters of each model [42,43].

## Authors contributions

GP and JE developed the mathematical model. GP implemented the model, run the simulations, performed the analysis and wrote the first draft. JE edited and wrote the revised versions. All authors read and approved the final manuscript.
